# Effect of total dissolved gas supersaturation and flow velocity on survival and swimming ability of juvenile *Schizothorax prenanti*

**DOI:** 10.1093/conphys/coad091

**Published:** 2023-12-07

**Authors:** Quan Yuan, Jun Du, Kefeng Li, Yuanming Wang, Ruifeng Liang

**Affiliations:** School of Energy and Power Engineering, Xihua University, Tuqiaojinzhou Road, Chengdu 610039, China; State Key Laboratory of Hydraulics and Mountain River Engineering, Sichuan University, Yihuan Road, Chengdu 610065, China; The Fishery Institute of the Sichuan Academy of Agricultural Sciences, Xiyuan Road, Chengdu 611730, China; State Key Laboratory of Hydraulics and Mountain River Engineering, Sichuan University, Yihuan Road, Chengdu 610065, China; State Key Laboratory of Hydraulics and Mountain River Engineering, Sichuan University, Yihuan Road, Chengdu 610065, China; State Key Laboratory of Hydraulics and Mountain River Engineering, Sichuan University, Yihuan Road, Chengdu 610065, China

**Keywords:** Flow velocity, Schizothorax prenanti, survival time, swimming ability, TDG supersaturation

## Abstract

Although developing large-scale hydropower cascades in the upper Yangtze River effectively improves the hydropower resource utilization, it produces total dissolved gas (TDG) supersaturation. In the flood season, the high level of TDG supersaturation (TDGS) frequently occurs in the downstream of dams, causing migratory fish to suffer from gas bubble trauma (GBT) and reducing their survival and swimming ability. Currently, there is a deficiency in particular approaches to evaluate the ecological hazard posed by TDGS on migratory fish as they traverse different flow velocities within their migratory routes. This study assessed the vulnerability of juvenile *Schizothorax prenanti* (*S. prenanti*) to GBT from the static setting to 9.0 BL/s during exposure to nominal levels of 100%, 110%, 120% and 130% TDG. The mortality occurs when the flow velocity surpasses 6.0 and 7.5 BL/s in 100% and 110% TDG levels, respectively. For fish exposed to 120% and 130% TDG levels, the relationship between survival time and flow velocity is an approximately inverse bell-shaped curve with increasing velocity. The optimal velocity of maximal survival time of juvenile *S. prenanti* is 3.0 and 4.5 BL/s in 120% and 130% TDG-supersaturated water. Both TDG level and flow velocity significantly affect burst swimming speed (U_burst_) and critical swimming speed (U_crit_). The cases involving GBT showed substantial declines in U_burst_ and U_crit_, exceeding 6.0 BL/s and TDG levels greater than 120%. The results may contribute to formulating a specific management strategy for hydropower operation during the migratory period and conserving vulnerable species in the Yangtze River.

## Teaser

This study evaluated the combined effects of TDG supersaturation and flow velocity on Schizothorax prenanti. TDG level and velocity had a significant impact on fish survival. TDG level had more negative impacts on Ucrit than Uburst, while velocity had comparable adverse effects on both Ucrit and Uburst of fish.

## Introduction

Hydroelectric dams are important global sources of electricity in terms of sustainability, but they bring some negative environmental impacts, including habitat fragmentation and total dissolved gas (TDG) supersaturation (Pleizier *et al.*, 2020a; Pelicice *et al.*, 2015; Zhang *et al.*, 2022). During the operation of a hydropower station, gas bubbles may be swept into the deep regions of the stilling basin amid flood discharge. In these high-pressure zones, the gas within the bubbles is compelled to dissolve into the water. As the water's depth increases, the gas’s solubility rises in tandem with the ambient pressure, enabling more air to transition from the bubbles into the water. This process amplifies the TDG level. The TDG supersaturation (TDGS) is produced by flood discharge in the operation of a hydropower station. The TDGS transports with the current to tens of kilometers of rivers away where the TDG at a high level remains for a long period due to the slow release of the gas from TDG-supersaturated water (Feng *et al.*, 2014). In these regulated rivers, long-term exposure to TDGS may cause fish to suffer from gas bubble trauma (GBT), producing lethal and sublethal effects, including immunosuppression and reduced swimming ability (Pleizier *et al.*, 2020a; Wang *et al.*, 2018; Liu *et al.*, 2011). Due to potential environmental risks, TDGS in the river basin with developing large-scale hydropower cascade has attracted extensive attention.

Some migratory species are forced to face TDGS and flow velocity challenges because their migratory period coincides with the flood season (Monk *et al.*, 1997; Johnson *et al.*, 2007; Stenberg, 2016). For example, Chinook salmon seems unable to move laterally to avoid areas with elevated TDG levels when it swims on the migratory route (Johnson *et al.*, 2007). Velocity barriers, such as falls, rapids and anthropogenic facilities, commonly occur. Examples of anthropogenic velocity barriers include culverts, some low-head or breached dams and technical fishways (Castro-Santos, 2006). Consequently, fish are sometimes even forced to cross the zones of high-velocity flow that exceed their maximum sustainable swim speeds during the upstream movements (Beamish, 1978). Studies conducted thus far, with respect to TDGS exposure, have been primarily under non-forced, lentic swimming conditions. The research domain lacks investigations in lotic circumstances (Pleizier *et al.*, 2023).

Juvenile *Schizothorax prenanti* (*S. prenanti*) is a reproductive potamodromous migratory fish species living in the upper Yangtze River, where many dams are constructed. During their annual spawning season from March to June, reproductive populations swim to the tributaries of the Minjiang and Dadu rivers, which are their breeding and spawning grounds (Li *et al.*, 2021; Shi *et al.*, 2022). The swimming ability of fish is a key factor for migration, and it may be influenced by TDG level and flow velocity. Critical swimming speed (U_crit_) provides a useful estimate of maximum aerobic swimming performance for fish. This variable can be used to assess migratory potential, while burst swimming speed (U_burst_) is an anaerobic exercise associated with predation and escape, by which fish pass velocity barriers during the migration (Castro-Santos, 2005; Harrison *et al.*, 2014). The connectivity between specific habitats is shrunk by dams. Fishway is a common structure that provides a route for migrating fish to pass through an artificial barrier. U_crit_ and U_burst_ of target species are variables critical to the fishway design (Beach, 1984), and *S. prenanti* is one of the target species for fishways designed in China (Shi *et al.*, 2022).

The coupled effect of TDG level and flow velocity may affect the survival of some migratory fish. These migratory fish that encounter varying flow velocities may adapt various swimming strategies to traverse them. Swimming patterns associated with physical activity, metabolic heat and blood pressure may inhibit or stimulate GBT formation, which may affect the TDG tolerance of fish (Wang *et al.*, 2016; Pleizier *et al.*, 2020b; Mcdonough and Hemmingsen, 1985). Previous studies showed that GBT may be chronic and work as a stressor that could reduce swimming ability. However, past studies were conducted without exposures to TDGS in flowing water prior to the swim performance tests (Wang *et al.*, 2017; Wang *et al.*, 2018). In the migration period, both TDG level and encountered flow velocity may represent an important environmental constraint to fish migration. The coupled effect of TDG level and flow velocity on the swimming ability has not been well clarified. This study investigated the synergistic effect of TDG level and flow velocity on survival and swimming ability in juvenile *S. prenanti*, which will provide representative data for TDGS-induced risk model for migratory fish during the flood season.

## Materials and Methods

### Ethics statement

The animal study proposal was approved by the Ethics Committee for Animal Experiments of Sichuan University. All experimental procedures were performed in accordance with the Regulations for the Administration of Affairs Concerning Experimental Animals approved by the State Council of the People's Republic of China.

### Experimental subjects

Juvenile *S. prenanti* were obtained from the Heima Hatchery in Hanyuan County, Sichuan Province, China. The *S. prenanti*'s mean weights and fork lengths were 5.1 ± 0.6 g and 7.8 ± 0.8 cm, respectively. All individuals were transferred from Heima Hatchery to the Laboratory of State Key of Hydraulics and Mountain River Engineering, Sichuan University, where the experiment was conducted. Before experiments, fish were held in aquariums with length, width and height of 1.2, 0.8 and 0.8 m, respectively. The aquariums were filled with equilibrium water at temperatures between 14.0°C and 15.5°C for 2 days. During the acclimation period, the water in the aquariums is continuously oxygenated and replaced with a third of the water twice a day. It should be noted that the fish used in the experiments were not fed during the experimental duration.

### Acute lethality experiment

The experimental apparatus included a system for generating TDG-supersaturated water and regulating TDG levels. Tap water and air were introduced into a pressure vessel via a pump and air compressor, respectively. Under high pressure, surplus air dissolved, leading to TDGS formation in the pressure vessel. An overflow tank was filled with high-level TDG-supersaturated water, while the other was filled with equilibrium water. Two valves were used to regulate the flow of high TDG and equilibrium water to produce water with the required TDG level for the experiment. Further details of the TDG-supersaturated water system are presented by Wang *et al.*, 2018.

**Figure 1:**
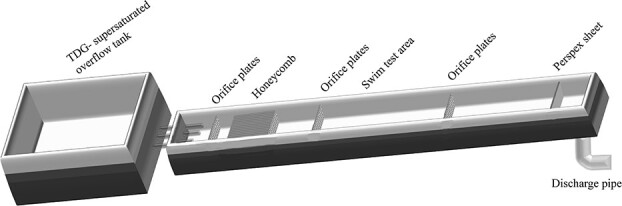
The geometric dimensions of TDG-supersaturated overflow tank and the open-channel flume consisting of orifice plates, honeycomb, swim test area and perspex sheet.

The experimental device consisted of a flume and an overflow tank placed at the front of the flume (Figure 1). The TDG-supersaturated water aquarium supplied water through pipes to the overflow tank where TDG levels were adjusted to the desired levels. A pump transferred the TDG-supersaturated water to an open channel flume with a cross-sectional size of 15 × 15 cm and a length of 4 m. This pump was also used to regulate the flow through the flume. The flume apparatus consisted of a flow regulating honeycomb, two pieces of orifice plates and a perspex sheet. The flow regulating honeycomb was established at the upstream end of the channel for generating a steady uniform flow. Two orifice plates were placed in the middle of the flume, which provided fish with a 1-m-long experimental zone. This zone was covered with plexiglass panels which were used to prevent fish from swimming up the flume during the trial. The perspex sheet was installed at the downstream end to adjust the 8-cm water depth.

An acute lethality experiment utilized a total of 1260 experimental fish. For each TDG level and assigned velocity, three replicate groups consisting of 15 fish each were established. Table 1 presents the details of juvenile *S. prenanti* used in this experiment. One-way analysis of variance (ANOVA) analysis, in conjunction with Turkey post hoc tests, ascertained any significant variations in mean fish length or weight among treatment groups. No significant differences were noted in length and weight among the treatment groups, except for the fish at 112% TDG and 4.5 body lengths per second (BL/s) group.

**Table 1:** Detailed information regarding juvenile *S. prenanti* utilized in the acute lethality experiment

TDG (%)	DO (mg/L)	T (°C)	Flow velocity (BL/s)	Length (cm)	Weight (g)
101 ± 1	8.78 ± 0.18	16.2 ± 0.1	Static	8.5 ± 0.5	7.5 ± 1.0
			1.5	8.4 ± 0.3	7.2 ± 0.5
			3.0	9.0 ± 0.9	8.7 ± 2.3
			4.5	9.4 ± 0.6	9.6 ± 1.5
			6.0	8.3 ± 0.2	7.2 ± 0.4
			7.5	8.1 ± 0.4	6.8 ± 0.7
			9.0	8.3 ± 0.2	7.2 ± 0.5
112 ± 1	7.46 ± 0.36	16.8 ± 0.2	Static	8.5 ± 1.0	7.6 ± 2.0
			1.5	8.5 ± 0.7	7.6 ± 1.5
			3.0	8.3 ± 0.7	7.2 ± 1.3
			4.5	10.4 ± 1.6^*^	12.4 ± 4.2^*^
			6.0	8.1 ± 0.3	6.8 ± 0.7
			7.5	8.2 ± 0.2	6.8 ± 0.3
			9.0	8.4 ± 0.5	7.3 ± 1.0
121 ± 2	7.61 ± 0.8	16.5 ± 0.1	static	8.1 ± 0.4	6.6 ± 0.8
			1.5	9.1 ± 0.6	8.9 ± 1.4
			3.0	8.2 ± 0.4	6.9 ± 0.8
			4.5	8.3 ± 0.6	7.0 ± 1.3
			6.0	8.8 ± 0.2	8.1 ± 0.4
			7.5	9.2 ± 0.2	9.0 ± 0.6
			9.0	8.4 ± 0.5	7.2 ± 0.7
130 ± 2	7.78 ± 0.15	16.4 ± 0.2	static	8.2 ± 0.4	7.0 ± 0.8
			1.5	9.0 ± 0.9	8.6 ± 1.9
			3.0	9.1 ± 0.6	8.8 ± 1.4
			4.5	8.0 ± 0.5	6.9 ± 2.9
			6.0	8.1 ± 0.3	6.7 ± 0.6
			7.5	8.0 ± 0.5	6.4 ± 0.9
			9.0	8.4 ± 0.3	7.4 ± 0.7

Date on TDG level, dissolved oxygen (DO) concentration, temperature, body length and weight are articulated as mean ± SE. Asterisks following the values indicate the results of the Turkey post hoc test; an asterisk denotes a significant disparity compared to other groups (*p* < 0.05).

In the experimental zone, the velocity was monitored with a wheel flow probe with an outer diameter of 30 mm. This probe (Loligo Systems AC10002, Denmark) offered high precision measurements of flow velocities in liquids with a flow range of 0.01 to 3 m/s. The TDG levels were monitored using the Point Four Tracker (Point Four Systems Inc., Canada). The TDG measurement tool underwent calibration before use. It achieved atmospheric pressure equilibrium upon exposure to air, and calibration was finalized by pressing the relevant button. Before TDG measurement, the probe was submerged in equilibrium water to obtain a 100 ± 1% reading. A stable reading was attained by gently oscillating the probe in the top layer of TDG-supersaturated water for a span of 3 to 5 minutes.

The acute lethality experiment had a duration of 96 hours. Experimental fish attached to the downstream net were examined. Fish that exhibited no substantial reaction to three consecutive touches with a small stick at 30-second intervals were presumed dead and promptly removed. The experimental fish's swimming behavior was continuously monitored. The abnormal behavior of fish was observed during the experiment, death time was recorded and the symptoms of GBT were photographed by a photomicroscope (Guangzhou Micro-Shot Optical Technology Co., Ltd, Guangzhou, China). The weight and fork length of juvenile *S. prenanti* were measured with an electronic balance and a dividing ruler, respectively.

### Swimming ability

Before the swimming test, juvenile *S. prenanti* were exposed to 100%, 110%, 120% and 130% TDG levels in the range of the static setting to 9.0 BL/s (static,1.5, 3.0, 4.5, 6.0, 7.5, 9.0BL/s) for 3.5 hours, because 3.5 hours is half of the lethal time in the worst scenario, i.e. 130% TDG and 9 BL/s. Dead fish were removed immediately and the remaining fish were tested for their swimming ability using a swimming tunnel respirometer (Loligo Systems SW10150, Denmark).

A total of 504 experimental fish were employed to explore the effects of TDG and flow velocity on the critical swimming ability. For each scenario, three replicate groups of six fish each were created, each exposed to specific TDG levels and flow velocities. The number of fish used to assess the impact of TDG and flow velocity on the burst swimming ability mirrored those in the critical swimming ability experiment. Detailed data on the experimental fish tested for swimming ability under each scenario can be found in Table 2. A one-way ANOVA analysis found no significant variations in fish body length and weight among different treatment groups, with exceptions being the groups at 110% TDG and 4.5 BL/s, 120% TDG and 1.5 BL/s and 130% TDG and 1.5 BL/s.

**Table 2:** In-depth information concerning the experimental fish tested for swimming capability under each scenario

TDG (%)	DO (mg/L)	T (°C)	Flow velocity (BL/s)	U_crit_		U_burst_	
				Length (cm)	Weight (g)	Length (cm)	Weight (g)
100 ± 1	8.54 ± 0.15	16.5 ± 0.2	static	9.1 ± 0.9	8.9 ± 2.2	8.3 ± 0.5	7.1 ± 1.1
			1.5	8.8 ± 0.6	8.1 ± 1.5	8.7 ± 0.4	7.9 ± 0.9
			3.0	8.8 ± 1.0	8.4 ± 2.5	9.0 ± 0.6	8.7 ± 1.6
			4.5	8.9 ± 0.9	8.5 ± 2.0	8.6 ± 0.8	7.8 ± 1.6
			6.0	8.4 ± 0.1	7.3 ± 0.2	8.3 ± 0.1	7.1 ± 0.3
			7.5	7.8 ± 0.1	6.1 ± 0.1	8.5 ± 0.1	7.5 ± 0.1
			9.0	8.7 ± 0.5	7.9 ± 1.0	8.6 ± 0.6	7.7 ± 1.2
111 ± 1	7.87 ± 0.29	16.6 ± 0.1	static	7.9 ± 0.7	6.4 ± 1.3	9.0 ± 0.7	8.5 ± 1.6
			1.5	8.0 ± 0.7	6.5 ± 1.3	9.1 ± 0.5	8.9 ± 1.2
			3.0	8.5 ± 0.9	7.5 ± 1.9	8.7 ± 0.1	7.8 ± 0.2
			4.5	9.7 ± 1.8	10.8 ± 1.7	10.0 ± 0.7^*^	11.1 ± 2.0^*^
			6.0	8.3 ± 0.2	7.1 ± 0.5	7.7 ± 0.6	6.0 ± 1.0
			7.5	8.0 ± 0.2	6.5 ± 0.4	8.2 ± 0.2	6.9 ± 0.4
			9.0	8.1 ± 0.7	6.7 ± 1.3	8.2 ± 0.7	7.0 ± 1.3
119 ± 2	7.61 ± 0.04	16.9 ± 0.2	static	8.2 ± 0.5	6.9 ± 1.0	8.2 ± 0.6	6.9 ± 1.2
			1.5	7.5 ± 1.2	5.8 ± 2.0	9.8 ± 1.0^*^	10.7 ± 2.7^*^
			3.0	8.2 ± 0.5	7.0 ± 1.0	8.4 ± 0.5	7.3 ± 1.1
			4.5	8.2 ± 0.6	6.9 ± 1.1	8.6 ± 0.3	7.8 ± 0.7
			6.0	8.6 ± 0.1	7.7 ± 0.2	8.9 ± 0.2	8.4 ± 0.4
			7.5	9.2 ± 0.3	8.9 ± 0.7	8.8 ± 0.3	8.1 ± 0.6
			9.0	8.2 ± 0.3	6.9 ± 0.5	8.5 ± 0.2	7.5 ± 0.4
131 ± 2	7.83 ± 0.04	16.7 ± 0.1	static	8.5 ± 0.2	7.4 ± 0.3	8.4 ± 0.7	7.4 ± 1.4
			1.5	8.0 ± 0.7	6.5 ± 1.2	10.4 ± 1.0^*^	12.3 ± 3.1^*^
			3.0	9.8 ± 2.1	11.2 ± 6.3	9.2 ± 0.7	9.1 ± 1.5
			4.5	7.0 ± 1.0^*^	4.8 ± 1.7	9.5 ± 0.1	9.8 ± 0.1
			6.0	8.1 ± 0.3	6.8 ± 0.6	8.2 ± 0.2	6.8 ± 0.4
			7.5	8.1 ± 0.3	6.6 ± 0.6	7.7 ± 0.4	6.0 ± 0.6
			9.0	8.3 ± 0.3	7.1 ± 0.6	8.4 ± 0.2	7.3 ± 0.4

Data on TDG level, DO concentration, temperature, body length and weight are articulated as mean ± SE. Asterisks following the values indicate the results of the Turkey post hoc test; an asterisk denotes a significant disparity compared to other groups (*p* < 0.05).

A swimming performance test was conducted in a variable speed swim tunnel respirometer with a control device and AutoResp 1 software. The swim tunnel had a size of 55 × 14 × 14 cm submerged in a 30 L buffer tank supplied with 16.7 ± 0.3°C aerated water, where the dissolved oxygen (DO) concentration level was above 7 mg/L, and the average TDG supersaturation was 102.0 ± 1.0%. Each group of fish was transferred into the swimming chamber with 0.5 BL/s flow for 20 minutes of acclimation. U_crit_ was calculated for individual fish using Brett's (1964) as follows:


(1) \begin{equation*} {U}_{crit}={V}_1+\left(t/T\right)\times {V}_2 \end{equation*}

where *T* is the allotted time to swim at each speed (15 minutes), *V*_1_ is the maximum speed the fish was allowed to travel for the whole time period (BL^−1^), *V*_2_ is the velocity increase (1 BL/s) and *t* (minutes) is the time the fish was allowed to swim at the final speed (minutes). A similar method was adopted to calculate U_burst_ to study the anaerobic swimming ability of fish. The water velocity was steadily increased by 1 BL/s every 20 seconds until the fish were exhausted in the swimming chamber.

All swimming speeds were represented as relative values (BL/s), derived by dividing the absolute speed by the body length.

### Statistical analysis

Data from tolerance experiments were processed by the computer-based fitting program (Prism, Graphpad version 5; Graphpad Software Inc). The influence of TDG and flow velocity on fish survival was assessed using time-to-event curves analysed by Cox proportional hazards regression. Hazard ratios offer a comparative measure of survival risk based on the reference event rate (Spruance *et al.*, 2004). For this research, hazard ratios were determined through the Mantel–Haenzel test to compare the survival curves of fish exposed to certain TDG levels under various flow velocities, against those in the static group.

The Cox proportional hazards model was used to determine the significance and contribution of TDG level and flow velocity to mortality. To estimate coefficient standard errors (SEs) and confidence intervals, the optimal Cox proportional hazards model was run using the coxph function with random intercepts specified as offsets. The hazard function expresses the likelihood of an incident happening at time *t* until which the subject has survived:


(2) \begin{equation*} {h}_1(t)={H}_0(t)\times \exp \left({b}_1{x}_1+{b}_2{x}_2........{b}_i{x}_i\right) \end{equation*}

where *H*_0_ is the baseline hazard, which is the hazard if all coefficients (*b*_1_...*b_i_*) are equal to zero, *x*_1_ … *x_i_* are covariates, which are the factors affecting the time of an event. The covariates' coefficients indicate the effect size of covariates.

A multiple linear regression model was used to determine the significance and contribution of TDG level and flow velocity to U_crit_ and U_burst_, respectively. The ‘lm’ function from the R environment was used to construct multiple linear regression models as follows:


(3) \begin{equation*} {h}_2(t)={b}_0+{b}_1{x}_1+{b}_2{x}_2........{b}_ix{}_i \end{equation*}

The significance level (*P*-level) was set to less than 0.05.

This study evaluated data distribution across various Cox proportional hazards models and applied the minimum Akaike information criterion (AIC) value to select the most suitable fitted model. The same process was employed to discern the optimal model among multiple linear regression models.

## Results

### Survival characteristics

**Figure 2:**
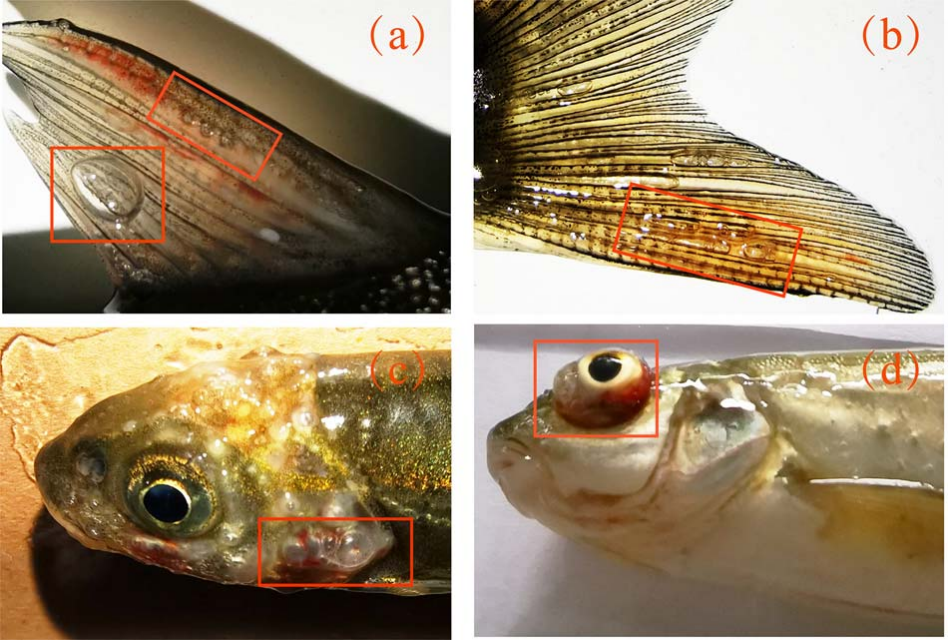
Examples of emphysemas in (a) the pectoral fin, (b) the pelvic fin, (c) the cutaneous and (d) the corneal of *S. prenanti* exposed to saturation levels of 120% and 130% TDG levels.

Some typical GBTs found in dead fish are illustrated shown in Figure 2. No GBT sign occurred among alive or dead *S. prenanti* exposed to 100% and 110% TDG levels under static and lotic settings. The cutaneous emphysemas were frequently observed in all major fins of fish acclimated to 120% and 130% TDG-supersaturated water under static settings. Occasionally emphysemas and gas emboli were observed in subcutaneous tissues over the head. The prevalence of the GBT sign decreased with the increasing flow velocity. Most fish died with no external signs of GBT greater than 6.0 BL/s.

**Figure 3:**
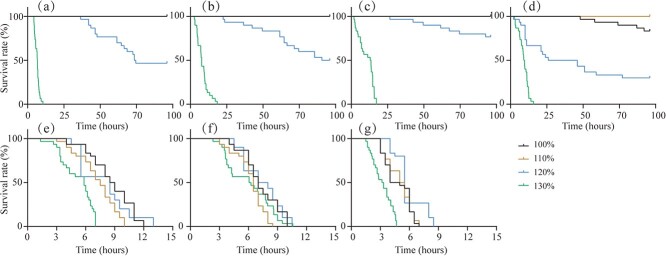
Survival curves of juvenile *S. prenanti* exposed to various TDG levels under (a) static condition and (b) 1.5, (c) 3.0, (d) 4.5, (e) 6.0, (f) 7.5 and (g) 9.0 BL/s. Each line represents the death time of 45 fish in three replicates.

The survival probability of juvenile *S. prenanti* exposed to TDG-supersaturated water with various flow velocities is plotted in Figure 3. No deaths occurred in 100% and 110% TDG-supersaturated water at a flow velocity of less than 4.5 BL/s. When the 120% TDG exposure period of 96 hours ended, the mortality of juvenile *S. prenanti* were 50%, 50% and 23% under the static setting, and 1.5, 3.0 BL/s, respectively. Figures 3e–g show that the survival rate of fish exposed to the 130% TDG level reached zero within 10.5, 18.4 and 17.3 hours under the static setting, 1.5, and 3.0 BL/s, respectively. When the flow velocity reached 4.5 BL/s, the mortality reached 16.7% at 96 hours in the 100% TDG level. Under 4.5 BL/s conditions, all individuals survived well in the 110% TDG level. 70% of fish died in the 120% TDG level within 96 hours, while all fish from the 130% TDG exposure group died within 15.8 hours (Figure 3d). Figures 3e–g show that all individual deaths came from different levels of TDG exposure groups within 12, 10 and 7 hours at 6.0, 7.5 and 9.0 BL/s. The TDG levels significantly affected the survival probability of juvenile *S. prenanti* in the flow velocity range of the static setting to 9.0 BL/s.

**Figure 4:**
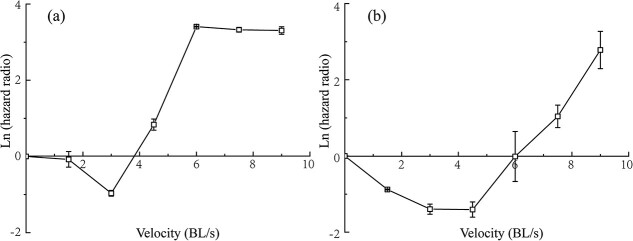
The logarithm of the estimated hazard ratio of juvenile *S. prenanti* exposed to 120% (a) and 130% (b) TDG levels at various flow velocities. The hazard ratio was calculated based on the survival time of fish under differing conditions, with the static condition serving as the baseline for comparison. For each TDG and velocity condition, three replicates were established.

**Table 3:** Estimated parameters for the Cox proportional hazards model of the lethal time for juvenile *S. prenanti* held under different TDG levels and flow velocities (*V*)

	Parameter	Coefficient	Exp (coef)	Standard error (SE)	z (Wald)	*p*	AIC
Model 1	TDG	0.06	1.06	0.004	13.18	**	6658
Model 2	V	0.41	1.51	0.02	17.68	**	6528
Model 3	TDG	0.06	1.06	0.01	11.87	**	6378
	V	0.37	1.45	0.02	16.64	**	

The coefficient and SE show the covariate effects on the lethal time for juvenile *S. prenanti*. *R*^2^ is the determination coefficient. Asterisks represent the significance of difference such that ^*^, ^**^ and ^***^ imply *p* less than 0.05, 0.01 and 0.001, respectively.

Fish exposed to the 120% TDG level displayed reduced mortality risk at flow velocities of 1.5 and 3.0 BL/s compared to the static condition. On the contrary, mortality risks escalated at flow velocities above 3.0 BL/s when contrasted with the static condition (Figure 4a). Survival curves analysis using the Gehan–Breslow–Wilcoxon test indicated a significantly lower survival probability at 6.0 (*p* < 0.01), 7.5 (*p* < 0.01) and 9.0 BL/s (*p* < 0.01) than under the static condition. In comparison to the static condition risk with the 130% TDG level, decreased mortality risks were noted within the flow velocity range of 1.5 to 4.5 BL/s. Conversely, mortality risks increased at flow velocities beyond 4.5 BL/s (Figure 4b). Based on the Gehan–Breslow–Wilcoxon test, the survival rate at 3.0 BL/s (*p* < 0.05) was significantly higher than the static condition, while the survival rate at 7.5 (*p* < 0.05) and 9.0 BL/s (*p* < 0.01) was significantly lower than that under the static condition.

The Cox proportional hazards model of the lethal time of juvenile *S. prenanti* is presented in Table 3. The optimal model for the survival time included both TDG level and flow velocity (Table 3), and it can be described as:


(4) \begin{equation*} LT=0.50\times e^{(-0.06\,\times\,TDGS\, \times\, 1\%\, +\, 0.37\times V)} \end{equation*}

### Burst swimming ability

**Figure 5:**
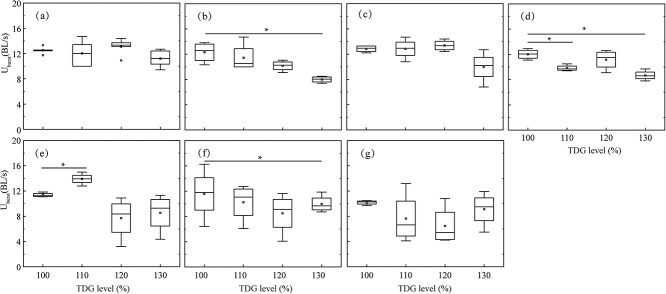
Box plot of U_burst_ for juvenile *S. prenanti* tested at various TDG levels under (a) static condition, (b) 1.5, (c) 3.0, (d) 4.5, (e) 6.0, (f) 7.5 and (g) 9.0 BL/s. The horizontal line within the box is the median, the ends of the rectangle are the first and third quartiles, and the range is the ends of whiskers. Asterisks indicate significance for *p* < 0.05.n = 18 for each flow velocity and TDG treatment.

Figure 5 shows the U_burst_ values of juvenile *S. prenanti* exposed to various TDG treatments from the static condition to 9.0 BL/s. Figures 5a, c and g show that, under the static condition, 3.0 BL/s and 9.0 BL/s, no difference in U_burst_ values was observed among juvenile *S. prenanti* among different TDG treatment groups (*p* > 0.05). Figures 5b and f show that, at 1.5 and 7.5 BL/s, the U_burst_ values of fish from the 130% TDG level group were significantly lower than those from the control group (*p* = 0.01 and *p* = 0.04). Figure 5d shows that, for fish at 4.5 BL/s, markedly decreased U_burst_ values were measured in 110% and 130% TDG exposure groups (for the 110% TDG level, *p* < 0.01; for the 130% TDG level, *p* < 0.01). For fish at 6.0 BL/s, the U_burst_ values were significantly increased in juvenile *S. prenanti* from 110% TDG treatment groups compared to those from 100% TDG treatment groups (*p* = 0.03) (Figure 5e).

**Figure 6:**
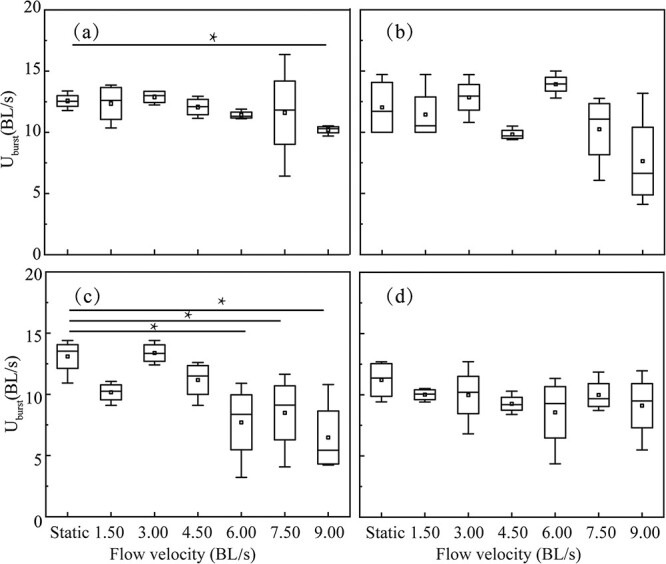
Box plot of U_burst_ for juvenile *S. prenanti* exposed to (a) 100, (b) 110, (c) 120 and (d) 130% TDG level at various flow velocities. The horizontal line within the box is the median, the ends of the rectangle are the first and third quartiles, and the range is the ends of whiskers. Asterisks indicate the significance of difference, i.e. * implies *p* less than 0.05. n = 18 for each flow velocity and TDG treatment.

Figure 6 shows that the U_burst_ values of juvenile *S. prenanti* experienced various flow velocities in a given TDG level. Figure 6a shows that, for fish placed in TDG-supersaturated water, the U_burst_ values were significantly reduced in juvenile fish from 9.0 BL/s groups compared with the static group (*p* = 0.01). Figures 6b and d show that there was no statistical difference in the U_burst_ values of juvenile fish from the different flow velocity treatment groups in 110% and 130% TDG exposure groups (*p* > 0.05). The U_burst_ value of juvenile *S. prenanti* from 6.0, 7.5 and 9.0 BL/s groups was significantly decreased compared to those in the static group, and a greater variability was observed in the U_burst_ value of juvenile *S. prenanti* from 6.0, 7.5 and 9.0 BL/s in 120% TDG exposure groups (for the 6.0 BL/s, *p* = 0.02; for the 7.5 BL/s, *p* = 0.03; for the 9.0 BL/s, *p* = 0.02) (Figure 6c).

The four selected models are presented in Table 4 to analyse U_burst_. The optimal model for U_burst_ values included both TDG level and flow velocity (*R*^2^ = 0.51). It was not improved by including the survival rate (*R*^2^ = 0.51). The relationship between U_burst_, TDG level and flow velocity was assessed using the multiple linear regression model as:


(5) \begin{equation*} U_{burst} = 21.89-8.49\,\times\, TDGS\, \times\, 1\% - 2.55\, \times\, V\end{equation*}

**Table 4:** Estimated parameters for linear models of U_burst_ for juvenile *S. prenanti* subjected to various TDG levels and flow velocities

		Coefficient	SE	*t* value	*p*	*R* ^2^	AIC
Model 1	Intercept	20.37	3.44	5.92	***	0.24	115
	TDG	−8.49	12.98	−2.85	**		
Model 2	Intercept	12.13	0.59	20.7	***	0.27	114
	V	−2.55	0.81	−3.14	**		
Model 3	Intercept	21.89	2.84	7.72	***	0.51	105
	TDG	−8.49	2.43	−3.5	**		
	velocity	−2.55	0.68	−3.75	***		
Model 4	Intercept	22.22	6.21	3.58	**	0.51	107
	SR	−0.18	2.96	−0.06			
	TDG	−8.62	3.29	−2.62	*		
	V	−2.58	0.95	−2.73	*		

The coefficient and SE indicate the covariate effects on U_burst_ for juvenile *S. prenanti R*^2^ is the determination coefficient. Asterisks indicate the significance of difference, i.e. ^*^, ^**^ and ^***^ imply *p* less than 0.05, 0.01 and 0.001, respectively.

### Critical swimming ability

**Figure 7:**
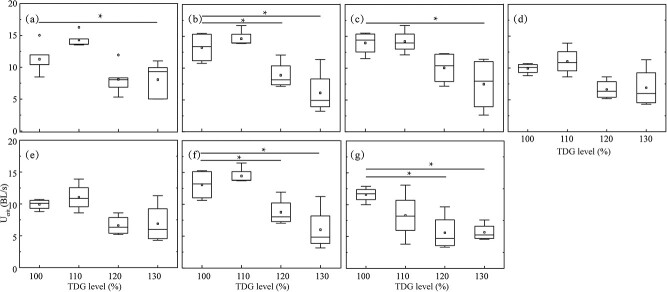
Box plot of U_crit_ for juvenile *S. prenanti* at various TDG levels under (a) static, (b) 1.5, (c) 3.0, (d) 4.5, (e) 6.0, (f) 7.5 and (g) 9.0 BL/s. The horizontal line within the box is the median, the ends of the rectangle are the first and third quartiles, and the range is ends of whiskers. Asterisks indicate the significance of the difference when *p* < 0.05. n = 18 for each flow velocity and TDG treatment.

Figure 7 shows U_crit_ for each fish group exposed to TDG-supersaturated water with various flow velocities. Figures 7a and c show that, under the static setting and 3.0 BL/s, the U_crit_ values were significantly decreased in 130% TDG exposure groups (for the static setting, *p* = 0.04; for the 3.0 BL/s, *p* = 0.04). Figures 7b, f and g show that U_crit_ values in juvenile *S. prenanti* exposed to 120% and 130% TDG levels significantly decreased at 1.5 (for the 120% TDG, *p* = 0.01; for the 130% TDG, *p* = 0.02), 7.5 (for the 120% TDG, *p* < 0.01; for the 130% TDG, *p* < 0.01) and 9.0 BL/s (for the 120% TDG, *p* = 0.01; for the 130% TDG, *p* < 0.01), respectively. Figures 7d and e show that, at 4.5 and 6.0 BL/s, there was no statistical difference in the U_crit_ value of juvenile *S. prenanti* among different TDG exposure treatment groups (*p* > 0.05).

**Figure 8:**
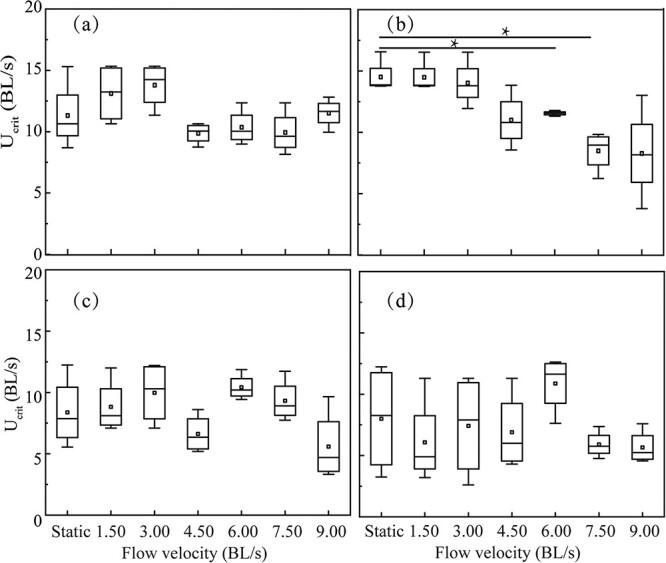
Box plot of U_crit_ for juvenile *S. prenanti* exposed to (a) 100, (b) 110, (c) 120 and (d) 130% TDG levels at various flow velocities. The horizontal line within the box is the median, the ends of the rectangle are the first and third quartiles, and the range is ends of whiskers. Asterisks indicate the significance of the difference. ^*^ and ^**^ imply *p* less than 0.05 and 0.01, respectively. n = 18 for each flow velocity and TDG treatment.

Figure 8 shows U_crit_ for each fish group exposed to various flow velocities in a given TDG level. Figures 8a, c and d show that no difference in U_crit_ values was observed among juvenile fish exposed to 100%, 120% and 130% TDG-supersaturated water with different flow velocities (*p* > 0.05). Figure 8b shows significantly decreased U_crit_ values of juvenile fish exposed to 110% TDG level at 6.0 and 7.5 BL/s (for the 6.0 BL/s, *p* = 0.03; for the 7.5 BL/s, *p* = 0.03).

The four selected models are presented in Table 5 to analyse U_crit_. Model 3, which includes both TDG level and flow velocity (*R*^2^ = 0.57, AIC 119), is the best fit to the data. This model was not improved by including the survival rate (*R*^2^ = 0.57, AIC 121). The relationship between U_crit_, TDG level and flow velocity was assessed using the multiple linear regression model as:


(6) \begin{equation*} U_{crit}= 29.56\,-15.99\, \times\, TDGS\, \times\, 1\% - 2.38\, \times\, V\end{equation*}

**Table 5:** Comparison of linear models of U_crit_ for juvenile *S. prenanti* experienced different TDG levels and flow velocities

		Coefficient	SE	*t* value	*p*	*R* ^2^	AIC
Model 1	Intercept	28.14	4.06	6.93	***	0.44	124
	TDGS	−15.99	3.52	−4.55	***		
Model 2	Intercept	11.18	0.89	12.59	***	0.13	137
	velocity	−2.38	1.23	−1.93			
Model 3	Intercept	29.56	3.68	8.02	***	0.57	119
	TDG	−15.99	3.16	−5.07	***		
	velocity	−2.38	0.88	−2.7	*		
Model 4	Intercept	31.84	8.05	3.96	***	0.57	121
	SR	−1.24	3.84	−0.32			
	TDG	−16.89	4.27	−3.96	***		
	velocity	−2.65	1.23	−2.16	*		

The coefficient and SE indicate the covariate effects on the lethal time for juvenile *S. prenanti*. *R*^2^ is the determination coefficient. Asterisks indicate significance of the difference. ^*^, ^**^ and ^***^ imply *p* less than 0.05, 0.01 and 0.001, respectively.

## Discussion

This study investigated the coupled effects of TDG level and flow velocity on survival probability and swimming ability (U_burst_ and U_crit_) of juvenile *S. prenanti*. In 100% and 110% TDG levels, flow velocity greater than 4.5 BL/s threatened the survival of the experimental fish, but in 120% and 130% TDG levels, the risk of mortality first declined and then increased with the increasing flow velocity. The optimal flow velocity for the mortality risk was affected by TDG levels. Increased flow velocity and elevated TDG level both significantly decreased the swimming ability. Specifically, the velocity dominated the influence of U_burst_ while the TDG level controlled the effect of U_crit_. No significant association was found between the survival rate and swimming ability of surviving individuals.

### Coupled effect of TDG level and flow velocity on survival characteristics of fish

The severity of GBT in fish may be affected by the interaction of TDG level and flow velocity. In static supersaturated water, GBT-induced abnormal behavior was found after TDG exposure. Losing buoyancy control resulted from hyperinflated swim bladders and lesion of the major fin. Opening their mouths frequently, flaring their gills and jumping out of the water indicated that bubbles in the gill filaments reduced the ventilation efficiency. A markedly different behavior was observed among juvenile *S. prenanti* challenging with high current velocity compared with unchallenging counterparts. With the same TDG level, frequently jumping out of the water and losing equilibrium were still observed among fish from high velocity groups. They exhibited less GBT-induced abnormal behavior than those from low velocity groups. The survival characteristics of fish suggest that the effect of TDG level on mortality decreased with the increasing flow velocity. TDG levels still significantly affected fish survival at a given flow velocity (Figure 3). A possible explanation is that increased mortality caused by excess gas in the circulatory system results in gas emboli blocking vascular flow to critical organs (Smiley *et al.*, 2011). Moreover, owing to the different rates of progression of GBT due to ventilation and gas diffusion of fish, their sensitivity to the lethal effects of GBT varied in terms of flow velocity variations. It is hypothesized that the combination of TDG level and flow velocity affects the rate of bubble production and development in the vasculature, changing the probability of fish survival.

To further analyse the coupled effect of TDG level and flow velocity on the survival characteristics of fish, survival time at various flow velocities was compared with that under the static conditions (Figure 4). Juvenile *S. prenanti* at 1.5 and 3.0 BL/s had a longer survival time than those under the static conditions in 120% and 130% TDG levels. This finding agrees with the results of Gray *et al.* (1983), indicating that Black bullhead are also more sensitive to TDG-supersaturated water under the static conditions than at 1.0 BL/s above the 130% TDG level. Like Black bullhead, *S. prenanti* exhibit excellent swimming ability. It is speculated that for active organisms, forced swimming induced by a low flow velocity is associated with decreased energy expenditure, causing less mortality compared to unforced exercise under a static setting. Similar observations were found in other species that forced swimming is associated with substantially lower energy costs compared with spontaneous activity. For example, Gilthead seabream (*Sparus aurata L.*) spent 2.5 times more energy adopting spontaneous swimming compared with forced swimming at a mean velocity of 0.5 BL/s (Steinhausen *et al.*, 2010). Boisclair and Tang (1993) investigated several species and indicated that this ratio between spontaneous swimming costs and those of forced swimming was in the range of 6.4 to 14.0. TDGS can lead to oxidative stress and cell damage, and energy is needed to recover from oxidative stress and lesion (Liu *et al.*, 2011). More energy is expended on GBT mitigation rather than swimming, which prolongs fish survival time. This speculation is supported by Bouck *et al.*'s (1976) finding that those individuals which were extremely active usually died before those remained relatively inactive in TDG-supersaturated water.

Our result shows that juvenile *S. prenanti* experience increased mortality under elevated flow velocities (greater and equal to 7.5 BL/s, 68% of U_crit_) than that in lentic conditions in 120% and 130% TDG-supersaturated water. It is worth noting that mortality occurs in equilibrium water at this flow velocity. One plausible explanation for these observations is that high-intensity swimming triggers a substantial surge in oxygen consumption, potentially leading to mortality due to relative hypoxia (Lee *et al.*, 2003). Increasing velocities also increase the ratio of anaerobic metabolism. Anaerobic exercise depletes energy (phosphocreatine, ATP and glycogen) and causes anaerobic metabolites (lactate) to generate and accumulate in vital organs such as muscle, liver and blood (Burgetz *et al.*, 1998; Wang *et al.*, 2016). An increase in the flow velocity accelerates the imbalance of metabolic expenditure to recovery and leads to physiological exhaustion. High flow velocities associated with anaerobic metabolism accelerate fish TDG-induced death. Other possible factors that could have contributed to rapid declines in survival at high flow velocities are as follows: (1) a faster rate of onset and progression of GBT due to increased ventilation (2) more prevalent and severe lesions of GBT caused by high flow velocity (3) a more difficult self-repair resulted from increased sharply swimming costs.

The Cox proportional hazards model showed that TDG level and flow velocity significantly affected the survival probability of fish (Table 3). For a given TDG level, the relationship between hazard ratio and flow velocity also exhibited an approximate inverse bell-shaped curve as the flow velocity increased. For instance, juvenile *S. prenanti* exhibited greater tolerance to TDG at 1.5, 3.0 or 4.5 BL/s than in lentic conditions whereas fish were more prone to TDG at 7.5 or 9.0 BL/s than under the static setting in 130% TDG-supersaturated water. This pattern of mortality associated with the flow velocity may be due to energy metabolism patterns. The cost of transport (COT), which provides an index for overall swimming efficiency, exhibited a similar typical inverse bell shape. A high COT is obtained at low swimming velocities and COT decreases to the minimum at an optimal swimming speed, and it increases sharply at greater velocities (Claireaux *et al.*, 2006; Tu *et al.*, 2011; Tu *et al.*, 2012). The results imply that mortalities of fish exposed to varying velocities are TDG-dependent. For example, when juvenile *S. prenanti* swam at 4.5 BL/s, there was a greater mortality risk for *S. prenanti* in the 120% TDG level and a lower risk in the 130% TDG level compared to those exposed to the corresponding TDG level under the static conditions. Similar results have been reported by Gray *et al.* (1983). They found that Cyprinus carpi were more sensitive to TDG-supersaturated water under lotic (1 BL/s, forced swimming) than lentic (non-forced swimming) conditions below 130% TDG. However, the opposite trend was observed above 130% TDG.

There are two explanations for the mechanism of GBT formation influenced by exercise. Pleizier *et al.* (2020b) suggested that exercise reduces the risk of bubbles blocking the cardiovascular system due to a decrease in oxygen content and an increase in blood pressure, thus reducing the likelihood of bubble growth in the cardiovascular system. Conversely, Mcdonough and Hemmingsen (1985) found that exercise may facilitate bubble nucleation through tribonucleation. The onset and progression of GBT may be affected by the characteristics of fish, such as anatomy or inheritable trait.

### Coupled effect of TDG level and flow velocity on fish swimming performance

Previous studies found that fish behavior may be influenced by flow velocities and TDG levels (Ji *et al.*, 2022; Shi *et al.*, 2022). In this study, fish were observed to swim randomly under static settings while they were found swimming in the same direction under lotic settings. In equilibrium water, all juveniles could maintain velocities of 1.5 and 3.0 BL/s without fatigue for more than 96 hours in the swim tunnel. It implies that the juvenile fish use sustained swimming mode to support moderate swimming velocities. The endurance time decreased with the increasing flow velocity, and half of the juvenile fish could not maintain 80 minutes endurance at 4.5 BL/s. They were swimming without fatigue for less than 15 minutes at 9.0 BL/s. It indicates that the juveniles adopt prolonged swimming speeds. Moreover, before fatigue, the juveniles in this velocity range tended to decrease steady undulatory swimming and increase an unsteady (burst and glide) locomotory gait as the flow velocity increased. Although fish in TDG-supersaturated water adopted a similar swimming pattern at a given velocity, they were exhausted in a shorter time.

The results of the multiple linear regression analysis revealed that TDG level and flow velocity significantly affected the U_burst_ (Table 4). Juvenile *S. prenanti* exposure to 130% TDG level for 3.5 hours may decrease the swimming ability. The swimming performance of the experimental fish in 120% TDG level did not show any difference in the U_brust_ compared with the control fish unexposed to TDGS (Figure 5). The U_burst_ values of fish exposed to more than 6.0 BL/s were significantly lower than those of fish under the static conditions (Figure 6). High flow velocities may compel fish to engage in anaerobic respiration, causing an accumulation of anaerobic metabolites such as lactic acid in muscles, liver and blood. This accumulation can diminish the subsequent swimming performance of fish (Reidy *et al.*, 2000). Figure 5 also shows that no significant differences were observed in the U_burst_ of fish exposed to 130% TDG level between lentic and lotic conditions. The possible reason is that high TDG levels decrease U_burst_ despite the flow velocity variation. The reduction in the anaerobic swimming performance with elevated TDG levels could stem from muscle contraction dysfunction resulting from TDG-induced muscle fiber damage (Chen *et al.*, 2023). Another possible explanation is that the reduction in the U_burst_ at a high TDG level might also be due to gas bubbles attaching to the fins, which are responsible for providing propulsion and balance (Lauder and Drucker, 2004).

The result showed that U_crit_ of juvenile *S. prenanti* significantly declined as the acclimation TDG level rose to the modest rate of 120% (Figure 7). There was no obvious trend between the effect of flow velocity and U_crit_ in 120% and 130% TDG-supersaturated water (Figure 8). This result suggests that the U_crit_ reduction is likely to be primarily due to increased TDG levels. The results also suggest that U_crit_ is more sensitive than U_burst_ to TDG levels, which agrees well with characteristics of juvenile *S. prenanti* and juvenile Chinese sucker studied by Wang *et al.* (2017). For two species, they observed a negative linear relationship between U_crit_ and U_burst_ with the TDG supersaturation level. They also found a greater downward trend in U_crit_ compared with U_burst_. U_crit_ represents the maximum aerobic swimming performance for fish and it more depends on ventilation efficiency than U_burst_. The decreased respiratory function due to GBT-induced gill damage may mainly cause such a difference. It is worth noting that swimming during U_crit_ tests is not purely aerobic, nor is swimming during burst swimming tests purely anaerobic. In the present study, oxygen uptake by the fish may have been impeded by the formation of bubbles within the gill filaments, which could have led to weakening aerobic exercise (Smiley *et al.*, 2011). Emboli occur in the gill and may block arteries that normally exhale carbon dioxide. A carbon dioxide increase raises bicarbonate and lowers pH which in turn corrodes the gills more severely (Michaelidis *et al.*, 2007). A second explanation for the greater reduction of U_crit_ is the reduction of function of the fish’s cardiorespiratory system due to the bubble embolism in arteries and veins in TDG-supersaturated water (Chong, 2022).

### Application and limitations

In the present study, juvenile *S. prenanti* experienced the same velocity zone in TDG-supersaturated water and in saturated water when they completed their migration. Previous studies indicated that juvenile *S. prenanti* can detect and avoid TDG-supersaturated water under laboratory conditions (Wang *et al.*, 2015). This means that the TDG level affects the flow velocity range experienced by fish by changing their migratory routes. However, a field study on adult Chinook salmon with vertical and lateral avoidance of TDG suggested that strong associations between migratory routes and the TDG level of the water or between the gas concentration and depth-use were not observed (Johnson *et al.*, 2007). The possible reason is that fish can easily detect TDG level gradient changes in small-scale laboratory water tank tests, while fish located in low TDG areas need to frequently move across the river, and this behavior of high energy consumption is contrary to the energy-saving principle for migration. The migratory route of fish is basically fixed and mainly depends on gene expression. The influences of environmental factors such as TDG levels are limited (Johnson *et al.*, 2007; Lennox *et al.*, 2022). Therefore, the range of flow velocities encountered by the migration of juvenile *S. prenanti* would not be altered by the influence of TDG levels.

The Yangtze River is fragmented by large dams, which have imposed two kinds of threats to migratory fish (Zhou *et al.*, 2015). Dams generally prevent upstream and downstream movements and TDGS is caused by dam operation, which have lethal and sublethal effects on fish. Fishways can occasionally help to mitigate the fragmentation caused by dams, though it remains challenging for migratory fish to evade TDGS exposure risk during the flood season. Previous studies on TDG tolerance were conducted under static conditions (Pleizier *et al.*, 2020a). However, static conditions are insufficient to determine the TDG threshold for migratory fish because extremely low flow velocities are difficult to be encountered in the migratory route since flow velocities provide the direction for migratory fish (Pelicice *et al.*, 2015). The results of the present study show that there are significant differences in fish survival time in TDG-supersaturated water between lotic and lentic conditions. The mortality risk model of the coupled effect of TDG level and flow velocity established in the present study can help determine survival thresholds during migration. Besides its lethal effect, this study reveals that the combined effect could decrease both U_brust_ and U_crit_, implying a diminished capacity to hunt and escape and a delayed arrival at spawning sites. More importantly, juvenile *S. prenanti* is the target species of fishways in China, and the decline of these two abilities implies that a larger proportion of fish fails to pass through fishways (Shi *et al.*, 2022). Further studies are needed to explore the threshold of TDG level regarding spawning activity and survival of *S. prenanti* eggs and fry. Based on these effects, strict TDG thresholds are expected to be established during migration periods.

Involuntary spills from high dams cause high flow velocities and elevated TDG levels in the flood season. Two strategies may minimize the potential risk of TDGS to fish exposed at high velocities. One is shifting spillway flows to different time periods, which could provide an opportunity to reduce TDGS and flow velocities (Witt *et al.*, 2017). The other is making operational changes at dams, which could direct the dissolved-gas plume to areas with a low probability of migratory fish. Fish have relatively certain migration paths. For example, migration for adult salmon is in close proximity to shorelines in large river systems (Standen *et al.*, 2004; Keefer *et al.*, 2006).

The present study has two inevitable limitations. This study provides the coupled effect of TDG level and flow velocity on the survival and swimming ability of juvenile fish. Thus, the results may not be applied to adult fish. However, investigating adult fish is necessary because they migrate upstream to spawn and undergo a return migration downstream to suitable habitats for feeding. Future studies on adult fish could allow us to estimate the impact of TDG level and flow velocity on adult migratory fish. The experiment of the present study is conducted in a laboratory, where fish exercising in open chambers are exposed to constant TDG levels and straightened flows. However, in hydraulic engineering practices, the TDG level always fluctuates with various flood discharges (Yuan *et al.*, 2021). Fish are studied under straightened flow conditions that probably do not often occur in nature. The forced swimming ability data adopted in this study may neglect the voluntary locomotory capacity and behavior of fish in the field. Thus, the results cannot be directly applied to estimate the migration rate of aquatic organisms in the river. Additional field tests on fish survival and swimming ability are needed to improve TDG risk assessment of migratory fish during the flood discharge.

## Conclusion

In this study, juvenile *S. prenanti* exercising in open channels were subjected for 96 hours to straightened flows ranging from lentic conditions to 9.0 BL/s with TDG levels of 100%, 110%, 120% and 130%. Fish survival was significantly impacted by TDG level and flow velocity. At flow velocities greater than 6.0 and 7.5 BL/s (45% and 68% of the critical swimming speed) in levels of 100% and 110% TDG, mortality results from exhaustion. The relationship between survival duration and flow velocity is inverse bell-shaped, with an optimal velocity between 3.0 and 4.5 BL/s in levels of 120% and 130% TDG levels.

The fish swimming test was conducted after exposure to TDGS and flow velocity for 3.5 hours, and the exposure scenarios were similar to those used in lethal experiments. The TDG level and flow velocity significantly impact the U_crit_ and U_burst_ of fish. The U_burst_ of fish may significantly decrease in 130% TDG-supersaturated water compared to that in saturated water, and no significant differences were found in 130% TDG-supersaturated water challenged with various flow velocities. The significant reduction in the U_burst_ of fish exposed to 120% TDG level and greater than 6.0 BL/s was observed. Despite the flow velocity variance, the U_burst_ decreases in 120% and 130% TDG levels. According to linear regression models, the TDG level has more negative impacts on U_crit_ than U_burst_, while the flow velocity has comparable negative effects on both U_crit_ and U_burst_ of fish.

## Acknowledgments

We deeply appreciate the support during the experimentprocess from Qianfeng Ji.

## Author Contribution

Quan Yuan (Methodology, Resources, Writing—original draft, Writing—review and editing, Funding acquisition), Jun Du (Writing—review and editing), Kefeng Li (Resources, Validation), Yuanming Wang (Conceptualization, Project administration, Funding acquisition) and Ruifeng Liang (Formal analysis, Data curation).

## Conflict of Interest

The authors declare that they have no known competing financial interests or personal relationships that could have appeared to influence the work reported in this paper.

## Funding

This research was financially supported by the Yangtze River Water Science Research Joint Fund (U2240212), the National Natural Science Foundation of China (52209100, 52179075) and the Science & Technology Fundamental Resources Investigation Program (2022FY100203).

## Data Availability

Data will be made available on request.

## Supplementary Material

Supplementary material is available at Conservation Physiology online.

## Supplementary Material

Web_Material_coad091
