# Supplementary material for: Effect of total dissolved gas supersaturation and flow velocity on survival and swimming ability of juvenile Schizothorax prenanti
Source: Conserv Physiol. 2023 Dec 7;11(1):coad091. doi: 10.1093/conphys/coad091 (PMC10709667; doi:10.1093/conphys/coad091)
Supplement: Web_Material_coad091 [file web_material_coad091.pdf]

Effect of total dissolved gas supersaturation and flow velocity on survival and swimming ability of juvenile *Schizothorax prenanti*

Quan Yuan [a,b], Jun Du [c], Kefeng Li [b], Yuanming Wang [b,*] and Ruifeng Liang [b]
[a] School of Energy and Power Engineering, Xihua University, Chengdu, 610039, China
[b] State Key Laboratory of Hydraulics and Mountain River Engineering, Sichuan University, Chengdu 610065, China
[c] The Fishery Institute of the Sichuan Academy of Agricultural Sciences, Chengdu, 611730, China

Table S1 Actual and relative swimming speed for juvenile *S. prenanti* tested at various TDG levels under static condition, 1.5, 3.0, 4.5, 6.0, 7.5, and 9.0 BL/s respectively.

TDG (%)	Flow velocity (BL/s)	Actual critical swimming speed (cm/s)	Relative critical swimming speed (BL/s)	Actual burst swimming speed (cm/s)	Relative burst swimming speed (BL/s)
100	static	103.8±13.9	11.6±2.5	103.8±4.3	12.5±0.6
	1.5	114.5±12.8	13.2±2.4	106.5±12.2	12.3±1.6
	3.0	121.0±6.4	13.9±1.8	116.0±6.5	12.9±0.5
	4.5	88.0±4.7	9.9±0.8	103.3±4.3	12.0±0.8
	6.0	87.7±11.3	10.4±1.4	94.3±1.7	11.4±0.3
	7.5	78.0±14.5	10.0±1.8	98.7±35.2	11.6±4.0
	9.0	101.3±16.3	11.6±1.2	87.3±3.3	10.2±0.4
110	static	115.3±6.2	14.6±1.2	106.8±12.9	12.0±2.1
	1.5	115.3±7.2	14.6±1.4	103.8±16.2	11.4±2.2
	3.0	117.8±3.9	14.1±1.9	111.5±14.1	12.9±1.6
	4.5	104.8±11.0	11.1±2.2	98.3±5.3	9.8±0.5
	6.0	96.7±3.7	11.6±0.2	106.7±3.4	13.9±0.9
	7.5	68.0±11.4	8.5±1.6	83.7±23.6	10.2±3.0
	9.0	65.5±25.7	8.3±3.8	63.3±32.8	7.6±4.0
120	static	69.0±22.7	8.4±2.5	107.2±9.7	13.1±1.3
	1.5	67.5±26.6	8.8±2.2	99.8±12.0	10.2±0.8
	3.0	83.0±24.8	10.0±2.5	111.8±8.2	13.4±0.9
	4.5	54.3±13.5	6.6±1.6	96.0±10.2	11.2±1.6
	6.0	89.7±8.8	10.4±1.0	69.3±29.8	7.7±3.3
	7.5	85.0±12.6	9.3±1.7	75.0±29.9	8.5±3.2
	9.0	46.5±25.7	5.6±2.9	55.7±27.8	6.5±3.1
130	static	70.3±23.8	8.3±2.9	93.8±8.3	11.2±1.4
	1.5	49.5±32.1	6.1±3.6	83.3±13.9	8.0±0.5
	3.0	67.0±31.6	7.4±4.2	90.5±15.7	10.0±2.4
	4.5	50.3±31.6	6.9±3.2	82.8±7.8	8.7±0.8
	6.0	89.3±22.2	10.9±2.3	70.3±26.1	8.6±3.0
	7.5	47.3±6.6	5.9±1.1	77.3±12.7	10.0±1.4
	9.0	47.0±11.6	5.7±1.4	77.0±24.3	9.1±2.7
